# Effects of Extirpation of the Spleen and Thyroid Gland

**Published:** 1845-04

**Authors:** 


*Effects of extirpation of the Spleen and Thyroid Gland.* By M. Bardeleben.—From M. Bardeleben's experiments, it results that the animals which survive the extirpation of the spleen appear to recover their health, and present no difference from those which have not undergone this operation. He never remarked that they were more voracious than other animals, though it has been said that voracity is generally produced. In no case was the organ regenerated, though Mayer of Bonn observed this. After removal of the thyroid gland the health was not sensibly affected; in one rabbit, however, the venereal appetite was considerably augmented,—a circumstance worthy of remark, seeing that it has often been asserted that removal of that gland abolished the venereal appetite. The animal deprived of both spleen and thyroid gland presented no change in any function. This fact is in opposition with the opinion of Tiedemann, that the lymphatic glands and thyroid body performed the functions of the spleen when that organ was extirpated. Finally, some physiologists have advanced the opinion that extirpation of the spleen produced augmentation of the venereal appetite, but abolition of the procreative faculty. M. Bardeleben satisfied himself of the incorrectness of this opinion, by breeding dogs which had both spleen and thyroid extirpation.—*Ibid, from Comp. Ren. des Seances de l'Acad. des Sci.*

